# Building implementation science capacity: Adaptation of I-Corps™@NCATS training for rapid fit-to-context discovery and designing for scale-up and sustainability

**DOI:** 10.1017/cts.2026.10691

**Published:** 2026-02-18

**Authors:** Elaine H. Morrato, Michael Bloom, Merly Thomas, Matthew Rivera, Nallely Mora

**Affiliations:** 1 Parkinson School of Health Sciences and Public Health, Loyola University Chicago, USA; 2 Center for Health Innovation and Entrepreneurship, Loyola University Chicago, USA

**Keywords:** Implementation science, Clinical and Translational Science Award (CTSA), workforce development, I-Corps@NCATS training program, designing for dissemination and sustainability

## Abstract

Clinical and Translational Science Award (CTSA) hubs must advance implementation science via innovative approaches to understand and develop strategies for overcoming barriers to the adoption, adaptation, integration, scale-up, and sustainability of evidence-based interventions, tools, policies, and guidelines. This special communication describes adaption of the I-Corps™@NCATS training program, a Lean Start-Up approach developed to advance commercialization of academic innovation, as a mechanism for building implementation science capacity at the Institute for Translational Medicine, a Chicago-based multi-institutional CTSA hub. Results from seven training cohorts (2021–2025) are presented (43 teams, 157 participants). In this five-week experiential program, teams conducted “customer discovery” interviews with stakeholders (mean = 23.8/team, SD = 5.6) to rapidly assess fit-to-context of their innovation and adoption requirements. Likelihood of recommending the program to a colleague was high (8.9, SD = 1.5; 1–10 scale, where “10” = “extremely likely”). Important adaptations were providing non-commercial use cases; defining “customers” in terms of stakeholders and partners; reframing commercial business model goals in terms of designing-for-dissemination-and-sustainability; and showing how the value proposition hypothesis is analogous to a research hypothesis being tested and validated with “customer discovery” data. Findings support that the modified I-Corps@NCATS training program provides flexible translational science skill-building to advance implementation science capacity among clinical and translational researchers.

Translation of new health knowledge and evidence to practice can take decades, with adoption often uneven, resulting in inequities and health disparities [1]. Proverbial “valleys of death” must be crossed to bring foundational basic science discoveries to clinical application and then to disseminate and integrate research findings more broadly into health policies affecting clinical decision-making and public policy [2]. A lack of relevance and fit with real-world demands, including practice constraints and competing interests within complex healthcare reimbursement models, often inhibits the diffusion of innovations reaching health professionals and the public. Patients, clinicians, and healthcare organizations often waste time and resources adopting and adapting solutions that are ultimately not scalable or financially sustainable, thereby reducing motivation to engage in the implementation of future solutions.

The National Institutes of Health (NIH) has sought solutions to address “why do so many interventions that have been well-funded, and found to be efficacious not become part of widespread care?” [3] When envisioning the future of research at higher education institutions, the National Academies noted that the most effective way to accelerate translation would be to begin with the end in mind and “anticipate the trajectory of the final innovative product right from the beginning of our programs in the most basic laboratories” [4]. The NIH has similarly advocated for training biomedical researchers to consider scale-up and implementation from the onset as a strategy for increasing the likelihood of health innovation being integrated into clinical practice [3,5].

Clinical and Translational Science Award (CTSA) hubs are funded by NIH to bring “more treatments to all people more quickly” and workforce development is a core element of these biomedical research capacity-building enterprises [6]. This special communication describes adaptation of the I-Corps™@NCATS training program[7,8], originally developed to advance commercialization of academic innovation, as a means to also build implementation science capacity at the Institute for Translational Medicine (ITM). The ITM is a multi-institutional Chicago CTSA program led by the University of Chicago and Rush University in collaboration with Advocate Aurora Health, Endeavor Health, the Illinois Institute of Technology, and Loyola University of Chicago. Results from seven training cohorts are presented, and the application for advancing implementation science is discussed.

## Designing for dissemination, implementation, and sustainability

The goal of health dissemination is to increase knowledge awareness and promote uptake, and it involves the active process of disseminating information to specific stakeholders and target audiences via their preferred communication channels using planned strategies [9]. Implementation science is the scientific study of methods to facilitate the adoption of that knowledge and evidence into routine practice with the goal of improving the quality and effectiveness of healthcare delivery and population health outcomes [10]. When compared to national investment on biomedical discovery and clinical research, pennies-on-the-dollar have been historically spent examining how academically-derived health innovation could be better disseminated and integrated into practice [9,11]. By one estimate, less than 10% of health researchers focused on dissemination activities, and only one-third involved stakeholders in the research process [1]. Today, each CTSA hub is now required to advance dissemination and implementation science via innovative approaches to understand and develop strategies for overcoming barriers to the adoption, adaptation, integration, scale-up, and sustainability of evidence-based interventions, tools, policies, and guidelines [6].

In recent years, there has been increasing emphasis on addressing implementation considerations earlier in the research process. Frameworks and tools have been developed to systemically address the challenge of designing for dissemination and sustainability (D4DS) and increasing compatibility between a health program, policy, or intervention and the context in which it is intended to be adopted [9]. The D4DS Fit-to-Context framework describes four phases - conceptualization, design, dissemination, and impact – that assist a research team in planning for what evidence, activities, partnerships, and structures will be necessary to maximize uptake and sustainable integration of a new health innovation (i.e., D4DS Planner Web) [9]. Design thinking necessitates that we examine the adoption and practice integration process from the perspectives of multiple stakeholders [12] and interactive fit-to-context exploration and validation are warranted to guide adaptations and increase relevancy [13]. The need for skill development in sustainability and scale-up methods has been identified as well as for advancing the science of engagement to assess stakeholder preferences while optimizing fit-to-context during the clinical and translational research process (i.e., Patient Centered Outcomes Research Institute- PCORI) [14].

## I-Corps™ – an evidence-based training program for designing for scale-up and sustainability of academically-derived innovation via commercialization

Launched in 2011, the Innovation-Corps (I-Corps) program is an accelerated team-based bootcamp version of Stanford University’s Lean Launchpad entrepreneur training for technology startups. It was developed in partnership with the National Science Foundation to magnify the societal impact of academically-derived innovation in science and technology [15]. The success of I-Corps in preparing scientists, engineers, and graduate students to extend their focus beyond the academic campus has been lauded by the U.S. Department of Commerce [16]. Since its inception, the I-Corps program has trained more than 7800 researchers evaluating over 3050 new technologies to determine product-market fit, with more than half of the teams launching startup companies to translate their technologies from the laboratory to the marketplace [15]. I-Corps is, at its core, a highly experiential learning training program grounded in the application of insights from over 100 discovery interviews with target customers, influencers, and economic decision makers to uncover the needs that drive their adoption behaviors. The impact of the I-Corps program on academic innovation in science and technology has been transformative in also advancing a translationally-minded workforce and in understanding that an academically successful innovation may or may not be commercially viable, i.e., scalable and sustainable, unless adaptation occurs (also referred to as pivoting) [7,8].

More than 40 percent of the NSF I-Corps teams sought to translate and commercialize technologies for the healthcare/medical sector [7]. Consequently, NIH also adapted the program for companies that received Small Business Innovation Research or Small Business Technology Transfer funding from NIH or the Centers for Disease Control and Prevention. The National Cancer Institute convened a similar training program using customer discovery and lean startup methods as a tool for moving evidence-based behavioral interventions into the marketplace [17]. The National Center for Advancing Translational Science (NCATS) adapted the training program for clinical and translational researchers and launched the program at ten CTSA hubs using a train-the-trainer model [7]. One of the authors (EM) was an originating member of the NCATS adaptation of the program, where she integrated the training into the CU Anschutz Medical Campus’ Research Evaluation and Commercialization Hub (REACH), funded by NIH to foster commercialization and entrepreneurship on the campus [18].

## I-Corps@NCATS at the Chicago Institute for Translational Medicine

The ITM participated in the three-year *National Expansion of the I-Corps@NCATS Program for Commercialization* initiative to establish entrepreneurship expertise and capacity within our Chicago-based CTSA hub [8]. Table 1 summarizes the program’s instructional design and translational science learning objectives as adapted for our local institutional context. The three phases of the training program are:

**Table 1.** Hybrid instructional design of the I-Corps™@NCATS program adapted for advancing implementation science

Instructional activity		Description – key concepts	Learning objectives
**Pre program** (weeks or months)xs	Outreach	Campus-wide town hall forums; individual department meetings. Over time, outreach has included word-of-mouth referrals.	To raise awareness of the benefits and skill development the I-Corps program offers *for health innovation design and implementation* To create a pipeline of teams primed for participation To ensure team member expectations match required time commitment (i.e., to increase the likelihood of successful completion)
	Team priming	Identifying the innovation team and training participants (typically, 2–4 members) Team orientation and expectations setting.	
	Training commitment	Selecting the best program timing for the team (i.e., Fall or Spring cohort) Advanced scheduling of 2–3 customer discovery interviews for Day 1 of training	
**I-Corps training program** (3 weeks)	In-person Interactive skill-building workshop: First 2 days	Instructional setting: “in-the-round” classroom to support collaboration and participation Instructor team: academics with entrepreneur experience; entrepreneurs with teaching interest Instructional style: a mixture of “radical candor” and peer-to-peer learning. A friendly shark tank. A series of skill development exercises: Value Proposition design centered on solving the customer’s most pressing “Pants on Fire” “Jobs, Pains, and Gains” *User experience journey modeling, workflow and Ecosystem mapping - help in identifying the target customer (adopter) in the context of all stakeholder influencers and decision makers affecting adoption* Customer discovery process – securing interviews; open-ended questioning; listening; iteratively synthesizing insights Identifying competing alternatives and customer metrics used to assess benefit	To refine and evaluate each team’s hypothesized Value Proposition using customer/stakeholder data *To learn a repeatable stakeholder engagement skills to identify and illustrate problems and opportunities for innovation can be rapidly and iteratively applied to the conceptualization, development, implementation, adaptation and sustainability of any health innovation (product, service, program, or policy)* To role model what teams can do next to advance the development of their innovation and to provide a warm-hand off to local technology transfer colleagues *and clinical and translational resources in implementation science*
	Off-line customer discovery: 4 weeks	“Get out of the building” and conduct customer and stakeholder interviews (target = 25+ interviews) Weekly 30-minute Office Hour check-ins with teams and instructors to discuss customer discovery challenges; what teams are hearing in their interviews; and implications for their Value Proposition hypothesis	
	In-person report out and next Steps workshop: Last day, week 5	Team Ignite-style presentations: customer discovery learning; value proposition implications; go, no-go, or pivot next steps Panel discussion: technology transfer and commercialization support available *Panel discussion: non-commercial translational pathways and supports available*	
**Post program** (6–12 months)	Seed Awards for teams who successfully complete the training program	Customer discovery ($2500) to expand and continue their customer discovery activities to further develop/validate their Value Proposition *Implementation ($25,000) to advance innovation implementation through a mentored approach*	To provide resources for further value proposition validation and sustaining momentum of translational implementation

*Note* : Italicized text indicates additions and adaptations to support implementation science research capacity building.

### Pre-program team identification and preparation

We conduct two training cohorts a year, spring and fall. Participant recruitment is a continuous process involving outreach, team priming, and commitment. Outreach across the five ITM-affiliated academic institutions includes newsletter advertisements, virtual town hall forums, professional social media platforms (i.e., LinkedIn), individual school and department meetings, and word-of-mouth referrals. Team priming is the process by which teams are vetted for training readiness. Readiness is defined as (1) having a health innovation with a clear user-adopter (i.e., “customer”) and a definable benefit (i.e., “value proposition” hypothesis); and (2) a team of researchers or practitioners working to advance the product. Innovation can range from commercializable (e.g., drugs, devices, diagnostics, and digital health) to non-commercializable products (e.g., health programs, services, and educational programming).

What makes our I-Corps@NCATS program unique is that learners are translational research teams, not individuals. Teams are generally two to three people, such as a more senior scientist/clinician and a more junior entrepreneurial leader (e.g., early career investigator or fellow/trainee) who can dedicate more time to leading the customer discovery interviews. The diversity of teams, therefore, promotes interdisciplinary collaboration and broad workforce development.

Prior to training, teams receive one-on-one orientation to preview program delivery and to affirm team commitment, including scheduling customer discovery interviews in preparation for day one of the course. Because the training program is highly experiential vs. lecture-only, it is critical that expectations are aligned with the instructional format.

### 5-week experiential learning sprint

The training begins with two half-days of in-person didactic lectures, interactive workshops, and initial two to three stakeholder interviews. The Supplement provides the course syllabus and pre-readings/videos. The course uses a business-oriented Value Proposition Design book as its instructional guide for creating, testing, and validating a testable value proposition hypothesis for why someone would want to adopt and use the proposed innovation [19]. The instructor team is comprised of academically-affiliated educators with entrepreneurial backgrounds and health entrepreneurs with a passion for mentorship and sharing their real-world learning. Feedback is direct – in the style of a real-time study section assessing strengths and limitations, or a la friendly “Shark Tank.”

Teams are introduced to lean start-up methods with a focus on problem-solution fit using customer discovery methods (see: https://venturewell.org/i-corps/team-materials/). Customer discovery involves iterative key-informant interviewing with stakeholders; each interview represents a mini-test of the value proposition hypothesis. Open-ended questioning and insights help teams understand who influences the adoption process and identify which jobs-to-be-done, problems-to-solve, or aspirations-to-gain represent the greatest value to incentivize trial and adoption. Teams aim for a pace of five interviews per week. Although uncomfortable for some, the pace is by design because it simulates the proactive outreach and entrepreneur hustle necessary for crossing the translational “valley of death.” During this time period, teams have virtual weekly check-ins and office hours with the faculty to check on progress, tackle roadblocks, and provide feedback.

On the final day teams’ re-group in person and present insights from their customer discovery and updated value proposition hypotheses. They are asked whether they will “go, no-go, or pivot” from their original idea based on what they learned.

### Post-program implementation seed funding

The purpose of the seed funding is to incentivize teams to maintain the translational momentum of their learning experience. Teams who complete training can apply for an ITM Customer Discovery Seed Award (up to $2500) to extend the customer discovery process. They have up to a year to use the funds to further validate their value proposition and business-sustainability model assumptions, thereby generating preliminary data for obtaining further funding.

Teams can also apply for an ITM Implementation Seed Award (up to $25,000) to receive mentorship on translating the I-Corps insights into actionable strategies and activities for implementing and translating their innovation into practice. Up to three teams are selected per year, and the seed program operates like a typical one-year pilot award program. Teams are paired with faculty mentors experienced with implementation in the context of the team’s area of innovation.

## Key adaptations for implementation science capacity building

A compelling business case and sustaining financial model are necessary to scale-up and maintain use of new health innovation once effectiveness is demonstrated – whether the intention is to commercialize for-profit or simply to incentivize adoption and health system integration. We know that how the innovation’s value proposition is framed, and the costs and economic impact associated with different implementation approaches influence policy makers and organizational decision-makers’ likelihood to adopt [11]. Approaching implementation like an entrepreneur encourages D4DS planning. I-Corps@NCATS training was adapted in the following ways to support implementation science capacity building.

1. **Repositioning the program’s target learner to include non-commercial use cases, thereby expanding its relevance and reach for translational researchers.** The University of Chicago Polsky Center for Entrepreneurship and Innovation is the academic home for technology transfer and the local instance of the NSF I-Corps program. Its focus is to advance entrepreneurship for students interested in pursuing a career in venture capital. To differentiate the ITM’s I-Corps@NCATS training, we targeted the program as an implementation science capacity-building initiative and integrated it into our Implementation Science Core. Table 2 lists an expanded list of translational research learners we target with our outreach, and the potential value that skill development in customer discovery and value proposition design might provide for each segment’s goals.
2. **Defining “customers” in terms of key stakeholders and partners, that is, terms familiar to clinical and translational scientists.** I-Corps uses business vernacular and focuses on identifying target customers and categorizing customers as users, influencers, recommenders, decision makers, economic buyers, and competitors. For researchers not seeking to commercialize their innovation, these terms can be confusing and/or off-putting. To bridge terms of art, we use the “Seven P’s” taxonomy for identifying stakeholders (Patient/Public, Providers, Purchasers, Payers, Policy Makers, Product Makers, and other Principal Investigator researchers) [12] as a mnemonic to guide stakeholder engagement and the discovery process. Participants map the decisional relationships between stakeholders (like “customers”) and identify the target segment most likely to adopt first. Stakeholders and their journey through the health service or medical product user experience is thus relationally visualized by teams to understand the problem, where it is occurring and how it is affecting the stakeholders [20].
3. **Reframing the business model goal of problem-solution fit as equivalent to the D4DS translational goal of fit-to-context.** Understanding the problem, where it occurs in the workflow and how to reframe innovation design contextually as a user experience is taught to the teams so they can conceptualize fit-to-context and implementation readiness [21]. The customer discovery interviews are thus a method to elicit the most important jobs to be done by the user, pains to solve, and motivating gains to achieve within their user experience context. These are the key ingredients for developing a compelling value proposition to support scale-up and sustainability. Participants learn to pivot, adapt, and focus their innovation design and dissemination planning to maximize fit.
4. **Defining the value proposition hypothesis as analogous to a research hypothesis to be tested and validated using the “customer discovery” stakeholder data**. A value proposition statement is a concise declaration that explains the specific benefits a product or company offers to its customers and why they should choose that company’s product or service over others. In translational science, clinical inertia and the *status quo* are often powerful “others” (i.e., competitors) impeding the adoption of innovations. Academic researchers are skilled in defining significance and benefits from a societal perspective (i.e., Translational Science Benefit Model). I-Corps, just like motivational interviewing used in clinical care, teaches the research team how to listen and take a person-centered approach to solicit perceptions, needs, and motivations and define motivating significance and benefits from an individual perspective. Table 3 provides illustrative value proposition statements resulting from the use of the I-Corps@NCATS method. Value proposition terminology is being increasingly used in healthcare and in the context of translational science [22–25].

**Table 2.** Target learners for the I-Corps™@NCATS program adapted for advancing implementation science capacity

Target learner - customer	Job-to-be-done goal	Value of customer discovery
**Traditional target segment**		
Academic scientist entrepreneur	Commercialize an academically derived innovation; secure seed funding	Evidence guiding market differentiation and go-no go and pivot business decisions
**Expanded customer segments**		
**Academic clinical & translational scientist**		
Intervention-program design	Begin-with-the-end in mind and increase the likelihood for implement-ability, adoption and sustainability in the real-world from the start	Pilot data informing fit-to-context conceptualization and design justification and evidence needed (e.g., primary and secondary endpoints) to incentivize implementation and support funding requests to demonstrate effectiveness
Intervention-program adaptation	Adapt an evidence-based innovation for a new setting, audience (for example, to address health disparity gaps)	Exploration of key barriers and facilitators affecting adoption in the new context that need to be addressed
Intervention-program sustainability	Sustain an evidence-based innovation after traditional development funding ends	Identifying motivating priorities necessary for sustaining the program (e.g., end users, health insurance payers, employers, government agencies)
Faculty career development	Secure an extramurally funded career development award requiring a training plan that advances translational science skills	A generalizable and citable evidence-based method endorsed by the National Institutes of Health, National Science Foundation and other funding agencies
Academic public health		
Public health student	Demonstrate competency in identifying and responding to community health needs (in order to graduate with a public health degree)	A flexible and citable evidence-based method for stakeholder engagement and demonstrating CEPH competencies in “Planning & Management to promote health,” “leadership,” and “systems thinking”
Public health practitioner	Facilitate professional and community sectors in translating knowledge to practice and enhance essential public health capacity	An evidence-based participatory approach for advancing scholarship of application and engaging stakeholders to collectively and sustainably address public health problems
Health services & healthcare delivery		
Health education	Disseminate health information to promote healthy behaviors and behavior change	A method for developing messaging and educational framing focused on an audience’s motivating priorities to increase the likelihood the education will be acted upon.
Quality improvement programs	Promote adoption of evidence-based practices to improve health outcomes	A method for understanding current barriers, influencers, and incentives affecting program adoption, in order to identify effective implementation strategies
Academic enterprise		
Education programs	Develop new certificates, degree programs, and other educational offerings that are financially profitable (sustainable).	A method for guiding instructional design development that meets the needs and motivations of today’s learners and satisfies a strong market demand
Administrative services	Deliver support services (e.g., for students, alumni, instructors, researchers) that deliver high satisfaction and meet budgetary constraints	A method for understanding-prioritizing unmet service needs and supporting service delivery optimization

*Note:* CEPH indicates Council on Education for Public Health.

**Table 3.** Example value proposition statements for clinical and translational science innovation

		Designing for scale up and sustainability examples		
Value proposition statement		Web-based cancer screening program	Chronic disease self-management mobile app	Mobile health program
Elements	Description	Cancer education (Jones LP, et al., 2020)	Chronic disease (Mora N, et al., 2021)	Obesity and mental health (Haddad R, et al., 2022)
For _________________ *(target customer)*	person most likely to adopt/use or purchase the health innovation	For African American faith-based organizations delivering health programs to their members…	For older adults ≥ 65 years with ≥2 chronic disease condition(s) …	For adults with obesity being treated for severe mental illness in community care settings…
Who ________________, *(the priority problem, job-to-be-done, or opportunity to address)*	primary motivation that drives the adoption (purchase) decision	… who want to offer evidence-based cancer education to improve awareness and promote screening	…who want to adhere to self-care activities and treatment	… who want to learn and engage in healthier behaviors and have easy access to emotional regulation support to reach their goals…
________________ *(innovation name)* *Is a ______________* *(innovation category)*	type of innovation helping the customer	Project HEAL (health through early awareness and learning) is a web-based training platform for community advisors	Chronic disease self-management app (CDapp) is a mobile application	Mobile health (mHealth) short-messaging systems (SMS)- based interactive obesity treatment approach (iOTA) is a series of SMS texts and weekly prompts to self-monitor weight and goal progress
that ________________. *(statement of benefit)*	specific value that the innovation promises to deliver to the customer	… that equips lay leaders with tools to educate peers with evidence-based health education and cancer awareness practices.	… that is easily comprehensible and serves as a primary disease management resource.	… that provides an increase in external support and accountability.
Unlike _______________, (*competing alternative*)	other ways the customer can meet their need today (including staying with the *status quo*)	Unlike one-time screening health fairs that don’t typically provide evidence-based education nor offer engagement continuity,	Unlike managing multiple disease-specific programs delivered through health providers or tailoring your own other wellness apps developed for the general population,	Unlike most interventions with some health behavior change delivered in community-based mental health care settings,
________________ *(innovation name)* *______________* *(statement of primary differentiation)*	reason-to-believe that the innovation will deliver its promise better than the alternative(s)	Project Heal is a culturally based, sustainable, web-based program that enables faith-based organizations to deliver cancer screening education to their congregants on demand.	CDapp improves self-confidence in managing multiple chronic disease conditions in an easily accessible way.	iOTA was co-developed and adapted for adults with serious mental illness, delivering tailored, scalable behavior change support and overcoming common barriers patients often face when adopting healthier behaviors.
How the I-Corps@NCATS training benefited the research project:		“The training revealed insights into how the business model canvas could be used as a tool to examine the commercial and/or scale-up potential of an evidence based intervention delivered in faith based organizations.”	“The customer discovery and value proposition methodology can be used as an alternative or complementary approach to formative research to generate valuable information in a brief period.”	It was a “novel stakeholder-centered method for designing health intervention for dissemination and sustainability, our results suggest adjustments to the current health coaching delivery model are needed for successful implementation in community setting.”

*Note:* Jones, et al.^2020^. Planning for community scale-up of Project HEAL: insights from the SPRINT initiative. *Health promotion practice*, *21*(6), 944-951. Mora, N., et al. 2023. Applying Customer Discovery Method to a Chronic Disease Self-Management Mobile App: Qualitative Study. *JMIR Form Res*. Nov 13;7:e50334. Haddad, R., et al. 2022. Using Innovation-Corps (I-Corps™) methods to adapt a mobile health (mhealth) obesity treatment for community mental health settings. *Front Digit Health*. May 27;4:835002.

## Evaluation results

Seven ITM I-Corps@NCATS cohorts, representing 43 teams and 157 participants, completed training between Fall 2021 and Spring 2025. Only two teams (4.6%) did not finish the program. More than 1,000 customer discovery interviews (median = 23.8 interviews/team, SD = 5.6) were completed by the last day of the workshop. Teams represented an array of affiliations: schools of medicine, health sciences, and public health; hospitals and health systems; and community/off-campus partners. Participants included faculty, research associates/staff, students, fellows, and residents, and industry mentors. Teams represented a spectrum of innovation (drugs, devices, diagnostics, AI solutions, health and education programs; biomedical research tools and services) across a range of translational research stages: basic science, clinical efficacy validation, and practice implementation.

Table 4 summarizes the post-training survey evaluation of the program as standardized using the national I-Corps@NCATS survey instrument (Supplement) [8]. Independent evaluators administered the online survey to all participants using Qualtrics XM within two weeks of the training. The mean survey response rate was 60.5%. Summative statistics were calculated using STATA NOW 18 SE. Open-ended questions were analyzed using rapid qualitative methods to derive relevant themes and supported by NVivo (version 12, QSR International) qualitative data analysis software. The study met the definition of quality improvement and was determined exempt by Loyola University Chicago’s Institutional Review Board (IRB #LU 217,689).

**Table 4.** Evaluation of the I-Corps@NCATS program adapted for rapid fit-to-context discovery

Training cohort	Fall 2021	Summer 2022	Spring 2023	Fall 2023	Spring 2024	Fall 2024	Spring 2025	Total
No. of teams	5	6	7	7	9	4	5	43
No. of participants	34	15	27	22	30	14	15	157
No. of survey respondents	15	8	17	12	20	12	11	95
Participant response rate (%)	44.1	53.3	63.0	54.5	67.0	85.7	73.3	60.5
**Quantitative evaluation – training outcome measures**								
**Customer discovery / stakeholder fit-to-context exploration**								
No. of interviews completed during training, mean (SD)	27.1 (2.3)	23 (7.3)	19.8 (1)	23.5(7.7)	25 (8.1)	22.5 (3.5)	25.6 (9.1)	23.8 (5.6)
Likelihood of continuing customer discovery interviewing (% “somewhat”/“extremely likely”)	100	87.5	88.2	75	67	85	82	83.5
**Perceptions of training experience (mean (SD); 1–5 scale, where 5 = “a great deal”)**								
“The training helped our team hone in our on product-market fit.”	5.0 (0.1)	4.8 (0.4)	4.5 (0.6)	4.4 (0.7)	4.3 (1.0)	4.7 (0.5)	4.4 (0.7)	4.6 (0.6)
“Presentations helped our team sharpen our pitch and/or more effectively market our innovation.”	4.7 (0.1)	4.5 (0.7)	4.2 (0.9)	4.0 (1.4)	4.0 (1.0)	4.9 (0.7)	4.1 (0.9)	4.3 (0.8)
“The team workshops helped me understand how to apply key concepts to my research project.”	4.8 (0.1)	3.9 (1.4)	4.4 (0.6)	4.3 (0.8)	4.2 (0.9)	4.8 (0.4)	4.5 (0.8)	4.4 (0.7)
**Perceived value of the training program (mean, SD)**								
Training experience in relation to time commitment (1–5 scale, where 5 = “outstanding”)	4.5 (0.2)	4.0 (1.4)	4.2 (0.9)	4.3 (0.9)	4.2 (0.9)	4.7 (0.5)	4.4 (0.7)	4.3 (0.8)
Likelihood would recommend program to a colleague (1–10 scale, where 10 = “extremely likely”)	9.1 (0.3)	8.8 (2.6)	8.3 (1.9)	8.7 (1.5)	8.1 (2.7)	9.6 (0.7)	9.5 (0.9)	8.9 (1.5)
Net promoter score *	78.6	54.6	47.6	58.0	48.0	92.0	90.0	67.0 (19.4)
**Qualitative evaluation - themes from open-ended survey questions**								
**Skill development**	**Design thinking** *“Approach to problem solving. Move from top down [technology driven] to [needs driven] bottom up.”* *“The interviews expanded our view of the problem and range of potential solutions.”* “If you have an issue that you are interested in exploring but don’t know where to start on, the program is great for helping you shape your vision”	**Innovation development** *“Clarified how to most effectively break our project into actionable interventions, pushed us to speak to individuals we wouldn’t have thought to reach out to and to use their insights to craft our vision”* *“It really helped us organize our thoughts .. of how our solution fits into the problem.”*						**Collaboration** *“Expanding my network to talk to non-clinicians and people outside of my field when implementing research projects”* “I didn’t have an entrepreneurial mindset before participating” *“It made me more excited about the possibilities of creating solutions for our problem.”*
**Training** **pain points**	**Time – “need to hit the ground running”** *“The only challenge we actually faced was the possibility for scheduling interviews.”* *“I really wish more information had been given earlier regarding number of interviews and short time window.”*				**Didactic delivery** “*The program is holistic, but it’s condensed, I’d like to do it again to better capitalize on the opportunities the process is providing.”* *“Talking to a mentor helped, but I wish that there was more explanations or examples throughout each presentation.”*			
**Opportunities to improve training satisfaction**	Program structure *“Add a week after week 2 that doesn’t include a [didactic] session but gives time for interview.”* Application of New Technology *“As an Entrepreneur-in-Residence team we used AI to help with our customer discovery and interview assessments which created incredible efficiency. We’d be happy to help teams in the future.”*				Pre-course preparation *“Ahead of the course…Develop an ecosystem map and study the roadblock that might be for talking to potential interviewees.”* Mentoring post-training “Potentially more opportunities to meet the mentor more than once a week, and then follow-up after program ends”			

*Note:* Data source: online evaluation survey administered within 5 days of the completion of the program. SD represents standard deviation.

*The Net Promoter Score (NPS) is a measure of customer satisfaction and loyalty established by Bain & Company. It is calculated as the percentage with “9” or “10” recommendation score minus the percentage who reported a “1–6” score. An NPS above 50 is excellent, and above 80 is world-class.

Participants rated the program strongly for perceived value concerning time commitment (mean = 4.3 (SD = 0.8), on a 1–5 scale where 5 = “outstanding”). Benefits included advancing their innovation itself as well as personal professional development. Participants reported a high likelihood for continuing the customer discovery process and for recommending the program to others (mean = 8.9 (SD = 1.5), on a 1–10 scale, where 10= “extremely likely”).

Qualitatively, participants valued skill development in design thinking, on how to approach innovation development, and in the entrepreneurial collaborative mindset fostered by the program. Findings are consistent with the national evaluation of the I-Corps@NCATS program that found skill development in several characteristics of a translational scientist: systems thinker, process innovator, boundary spanner, team player, and skilled communicator, intellectual humility, cognitive flexibility, and skilled communicator [8]. Participants reported the benefit of learning a repeatable process for validating problem-solution fit, as also captured in testimonial videos (see: https://bit.ly/44xEwB1 and https://bit.ly/4nxtyE1).

The main pain points of the training program are the challenge of completing the discovery interviews, given its condensed sprint format. In response to this feedback, the program’s duration was lengthened from four to five weeks, and participant satisfaction improved (see Fall 2024 and Spring 2025 results). The program is also a departure from traditional terminology, and participants continue to request more case examples. Over time, a bibliography of I-Corps case applications to implementation science has been accruing [23,24,26–28].

Participants also reported that they struggled with securing resources to maintain customer discovery and translational momentum after completing the training. This feedback informed the addition of the Customer Discovery and Implementation Seed Grants as post-training funding opportunities to extend value proposition validation and implementation fit-to-context application.

## Reflection

Further adapting the evidence-based I-Corps program to advance translational science earlier in the research process and to strengthen implementation science capacity is a logical extension to meet workforce demand for D4DS skills. Training in design thinking and entrepreneurship is increasingly being integrated into medical curricula [29]; new educational frameworks and competency models are emerging [30]; and promotion and tenure guidelines are evolving [31]. Hospitals and health systems are investing in innovation centers and strengthening workforce capacity to develop human-centered design solutions to improve healthcare delivery and patient care.

We embraced entrepreneurism ourselves in adapting, scaling, and sustaining the I-Corps@NCATS program within the Chicago ITM ecosystem. We focused first on how the program could best demonstrate value. National dissemination of I-Corps@NCATS helps to achieve NCATS’ translational science goals (“gain” creation). We envisioned the program as foundational skill- and workforce capacity building within the ITM’s Implementation Science Optional Module (a local “job-to-be-done”). The ITM program did not replace traditional entrepreneurship programs, e.g., offered through the business school, but rather extended access and tailored the format and content to fit the academic rhythm and learning-style preferences for academic biomedical researchers (“pain” relief).

Next, we adapted the program to meet the needs of both translational scientists and practitioners (our CTSA client “customers”) to ensure sufficient “market demand” to sustain the program. We embraced the principle of “make a little, sell a little” to start on a small scale, evaluate, iterate, and grow. Our own “customer discovery interviews” with participants of our first two training cohorts validated our approach. We fine-tuned the program based on evaluation results and closely tracked program satisfaction. Sustained outreach/advertising across five ITM-affiliated institutions resulted in a sustained pool of five teams *in queue* contemplating participation. Team orientation, which reinforces program intensity and time commitment involved, helped establish realistic expectations and allowed teams to come prepared, a key factor contributing to high completion rate. As program awareness grew, so did the variety of participating teams, including non-commercializable innovations at different stages of dissemination and implementation, similar to training supported by the National Cancer Institute [17].

Lastly, several strategies were taken to sustain the program after the dissemination train-the-trainer grant ended. First, the I-Corps@NCATS program is now funded, and institutionalized, within the ITM’s implementation science core. It is funded through 2027 as a research capacity-building training program. Methods have been incorporated into federally-funded implementation research [23]. Most recently, an ITM-sponsored professional certificate program on designing for implementation and sustainability launched in fall 2025 grounded methodologically with the D4DS Fit-to-Context framework [9] and is teaching the I-Corps@NCATS customer discovery methodology.

## Further investigation

Program assessment was limited to short-term outcomes based on survey feedback solicited immediately following completion of the training program. Outcomes assessed were skill acquisition, attitudes and program satisfaction. As part of the logic model for the ITM’s implementation science core, further program evaluation is planned to assess long-term outcomes (including, sustained stakeholder engagement and value proposition validation throughout the translational process of design, demonstration and dissemination) and impact (including, frequency and magnitude of scale-up of the innovation). The Practical, Robust Implementation and Sustainability Model (PRISM) [32] and Translational Science Benefits Model [33] will be used as frameworks for assessing implementation and impact of the innovation workshopped via the I-Corps@NCATS program. Focused exploration of the I-Corps methodology as a means for building translational science research capacity will continue to be evaluated.

## Summary

The I-Corps@NCATS training program has proven a flexible training approach to help increase the reach of implementation science design skills at our Chicago ITM. Its skill building in value proposition design and business entrepreneurship concepts are easily adaptable and transferable for skill building in rapid fit-to-context exploration and design thinking necessary for implementation, scale-up and sustainability planning.

## Supporting information

10.1017/cts.2026.10691.sm001 Morrato et al. supplementary material Morrato et al. supplementary material

## Supplementary material

The supplementary material for this article can be found at https://doi.org/10.1017/cts.2026.10691.
