# Supplementary material for: Building implementation science capacity: Adaptation of I-Corps™@NCATS training for rapid fit-to-context discovery and designing for scale-up and sustainability
Source: J Clin Transl Sci. 2026 Feb 18;10(1):e56. doi: 10.1017/cts.2026.10691 (PMC13040300; doi:10.1017/cts.2026.10691)
Supplement: Morrato et al. supplementary material [file S2059866126106918sup001.docx]

Supplemental Materials

Building Implementation Science Capacity: Adaptation of I-Corps™@NCATS Training for Rapid Fit-to-Context Discovery and Designing for Scale-Up and Sustainability

Contents

[I. Course Syllabus 2](#_Toc214537815)

[II. Course Evaluation 7](#_Toc214537832)

[II.A Survey Instrument 9](#_Toc214537833)

[II.B Observational Protocol and Fidelity-of-Implementation Checklist 17](#_Toc214537834)

[II.C Interview guide for instructors and administrators 18](#_Toc214537835)

# Course Syllabus

Spring 2025 Cohort

| **Schedule:** | *Opening Workshops*: | Thursday/Friday, February 6 & 7, 2025 (in-person) |
| --- | --- | --- |
|  | *Closing Workshop*: | March 7, 2025 (in-person) |
|  | *Office Hours*: | Mandatory each week (remote) |

**Course Management:** Slack will be used extensively.

**Course Description:**

Customer Discovery is an iterative process of interviewing potential customers and stakeholders to understand problems and needs in the market and in society. These interviews lead to real-

world learnings and insights that validate or invalidate key components of the business model, often leading to pivots or refinements.

This course will provide teams with a hands-on learning experience utilizing Customer Discovery and successfully transferring knowledge into products, processes and services that benefit society. The entire team will engage with industry. You and your team will spend your time talking to and learning from customers, partners and competitors, and learning how to deal with the chaos and uncertainty of implementing innovative solutions and creating ventures.

This course is about getting out of the building. It is not about the lectures. You will be spending a significant amount of time outside the building, talking to customers and testing your hypotheses about what they want in products and services. We will spend our limited class time on what you learned from talking to customers, not what you already knew coming into the course.

**Class Culture**

This program was developed by entrepreneurs. We have limited time, and we push, challenge, and question you to make maximum use of our limited time together. We will be direct, open, and tough – just like the real world. We hope you can recognize that these comments aren’t personal, but part of the process. We also expect you to question us, challenge our point of view if you disagree, and engage in a real dialog with the teaching team.

**Interviews: 30+ Total**

As a team, you are required to interview ***at least 30 people*** during the program and log your interviews. This is a key metric used by the faculty to evaluate your progress. Planning is critical to your success; please make your interviews the top priority.

**Presentations: Opening, Weekly Office Hours, and Closing Session**

We will be using a PowerPoint template for reporting weekly deliverables. That template will be critical to keep the faculty up to date on your activities.

**Final Deliverables**

In addition to the 30 interviews, each team will produce and present the following: *Lessons Learned*

presentation (~10 minutes)

**Pre-Course Deliverables**

**Homework to be completed prior to Opening Day:**

**Required Tasks:**

1. Please **schedule two or three interviews BEFORE Day1!** These will be conducted during the “Out of the Building” hours on Thursday, Feb. 6, 2025
2. Contact and confirm **10** additional interviews to be conducted during Week 2.
3. Prepare and upload intro presentation - 3-minute presentations (Templates for slides and sample presentations will be available in Slack).

**Slide 1**: Team member names and photos; Team name and logo; business model hypothesis: who is the customer? What is the product, service or intervention? What is your value proposition and why would they buy/need it?

**Slide 2**: Tell us about your service or product idea (for example, identify key features)

**Slide 3**: Who is your target customer? And why do they care?

**Slide 4**: Who are your direct competitors? That is, who is currently serving your target customer? How are you different?

**Slide 5**: What are you most uncertain about your business model hypothesis? Tell us about the 10 customer interviews you have scheduled or proposed for Week 2. **Upload your presentation to Slack by 8:00 AM CST on 2/6/25. Use this naming convention: TeamLeadName_Date, e.g. Smith_February2025**

## Read and watch:

1. Read T*alking with Humans* and [giffconstable.com/2012/12/12-tips-](http://giffconstable.com/2012/12/12-tips-for-early-customer-development-interviews-revision-3/) [for-early-](http://giffconstable.com/2012/12/12-tips-for-early-customer-development-interviews-revision-3/) [customer-development-interviews-revision-3/](http://giffconstable.com/2012/12/12-tips-for-early-customer-development-interviews-revision-3/)
2. These short videos provide helpful tips and examples for preparing for your customer interviews:

[Pre-Planning Pt. 1](http://vimeo.com/groups/204136/videos/75308828) (4:55)

[Interviews Pt. 1](http://vimeo.com/groups/204136/videos/75535337) (5:40)

[Interviews Pt. 2](http://vimeo.com/groups/204136/videos/75536337) (3:49)

[Asking the Right Question](http://vimeo.com/groups/204136/videos/74338298) (2:37)

[Assuming you know what the customer wants](http://vimeo.com/groups/204136/videos/76175907) (1:56) [The](https://www.youtube.com/watch?v=KZYntTU3JW4) [Rules for Customer Interviews](https://www.youtube.com/watch?v=KZYntTU3JW4)

[Good and Bad Examples of Customer Interview Questions](https://www.youtube.com/watch?v=Cl9vMBFHIf0) [What is Design Thinking?](https://www.youtube.com/watch?v=ldYzbV0NDp8)

1. Read the following peer-reviewed articles:

[Designing for Dissemination and Sustainability to Promote Equitable Impacts on Health](https://loyolauniversitychicago.sharepoint.com/sites/GroupI-CorpsLoyola/Shared%20Documents/Forms/AllItems.aspx?id=%2Fsites%2FGroupI%2DCorpsLoyola%2FShared%20Documents%2FI%2DCorps%20articles%2FDesigning%20for%20Dissemination%20and%20Sustainablity%5FARPH%5FDEC%202021%2Epdf&parent=%2Fsites%2FGroupI%2DCorpsLoyola%2FShared%20Documents%2FI%2DCorps%20articles&p=true&ga=1)

[Customer discovery as a tool for moving behavioral interventions into the marketplace](https://pmc.ncbi.nlm.nih.gov/articles/PMC7184906/pdf/ibz103.pdf)

**Opening Workshop - February 6, 2025: Day 1**

**Opening Day – Schedule *(subject to change)***

## 8:30 am Check-in

*Coffee and a light breakfast are served*

## 9-9:30 am Introduction to I-Corps

*Program goals and faculty*

## 9:30-10:15 am Team Presentations

**10:15-10:30 am Slack and Uploading Deliverables**

**10:30-10:45 am Break**

### 10:45-Noon Workshop #1: Testing and validating your value proposition

**Teamwork:** Hypothesize your Value Proposition. 1-on-1 discussions with Instructors

**Noon-1:30 pm Working Lunch** *(Lunch Provided)*

### Workshop #2 - Customer Discovery Process

- *Identifying Target Customers*
- *Customer Types*
- *Interviewing*

**Teamwork:** TBD

**1:30 pm Out of the building for interviews** (2-3 customers)

**Opening Workshop – February 7, 2025: Day 2**

**Homework to be completed prior to Day 2:**

**Read and review:**

[**https://steveblank.com/2013/11/08/a-new-way-to-look-at-competitors/**](https://steveblank.com/2013/11/08/a-new-way-to-look-at-competitors/)

**Watch:**

[Understanding the Problem (the right way)](http://vimeo.com/groups/204136/videos/76173388) (3:22) [Customers Lie](http://vimeo.com/groups/204136/videos/76176674) (2:37)

[The Distracted Customer](http://vimeo.com/groups/204136/videos/73715398) (3:12) [Engaging the Customer](http://vimeo.com/groups/204136/videos/76174533) (3:37) [Customer Empathy](http://vimeo.com/groups/204136/videos/73714461) (2:25)

[The User, the Buyer & the Saboteur](http://vimeo.com/groups/204136/videos/73673203)

**Day 2 – Schedule *(subject to change)***

**8:30 am** *Coffee and light breakfast served*

## 9:00-11:00 am Debrief interviews: Why are we doing this?

### Workshop #3: Business Models Basics

- *Problem-Solution Fit*
- *Product-Market Fit*
- *Value Proposition Hypothesis Revisited*
- *Ecosystems and the 7 Ps*

**Teamwork Activity:** *Draw your Ecosystem Map*

## 11:00-11:15 am Break

**11:15-12:45 pm Working Lunch** *(Lunch Provided)*

## Workshops – Expanding Customer Discovery with Maps

### Workshop #4a: Journey Maps

*Teamwork: Draw your Journey Map. 1-on-1 discussions with instructors.*

### Workshop #4b: Workflow Maps

*Teamwork: Draw your Workflow Map. 1-on-1 discussions with instructors.*

**12:45 – 1:45 pm Final Session -** *Define your customer discovery plan (next 3 weeks).*

*1-on-1 discussions with instructors.*

# Office Hours during the intervening weeks

You are required to meet with one of the faculty once per week for a period of 20 - 30 minutes. You will have the opportunity to sign up with individual faculty. Week 2 February 10-14, Week 3 February 17-21, Week 4 February 24-28

**March 7, 2025: Closing Workshop**

**Homework to be completed prior to Closing Session: Required Tasks:**

Prepare and upload Final presentation – 10-minute presentations, 5-minute Q&A (Templates for slides and sample presentations will be available in the Drop Box).

**Upload your presentation to Slack by 8:00 AM CST on March 7, 2025. Use this naming convention: TeamLeadName_Date, e.g. Smith_March 2025**

## Closing Session – Schedule

**8:30 am** *Coffee and light breakfast served*

## 9:00 – 9:10 am Welcome Back & Review

**9:10-11:45 am Team Presentations – Tell your Customer Discovery Story**

- Each team presents for 10 minutes, followed by 3 minutes of instructor feedback
- Slide templates will be provided in advance to include:

**Slide 1:** Team member names and photos; Company name and logo; number of interviews completed

**Slide 2**: Top 3 insights from customer discovery

**Slide 3**: Updated business hypothesis (customer + value)

**Slide 4**: Ecosystem Map

**Slide 5**: Journey or Workflow Map

**Slide 5**: What still needs to be validated?

**Slide 6**: Next steps for your team: Go. Pivot, or No Go.

**11:45 am-1:15 pm “What’s Next?”** *– (Lunch Provided)*

**Panel: Navigating Local Resources**

ITM/Implementation Science /NSF I-Corps, SBIRs

- CBC HITES Representative
- Loyola University
- MATTER representative

## 1:15–2:45 pm Optional Office Hours with Faculty

This project is supported by the National Center for Advancing Translational Sciences (NCATS) of the National Institutes of Health (NIH) through Grant Numbers UL1TR002389, KL2TR002387, and TL1TR00238 that fund the Institute for Translational Medicine (ITM).

# Course Evaluation

The original evaluation of the I-Corps@NCATS program occurred as part of the 2017-2019 train-the-trainer supplement award involving 10 Clinical and Translational Science Award (CTSA) hubs (grant no. NIH/NCATS UL1TR001417-02S). Program evaluation examined results from eight training cohorts (62 teams and 150 individuals). The evaluation survey instrument was adapted from the National Science Foundation’s I-Corps survey instrument for a clinical and translational science context.

Nearing K, Rainwater J, Neves S, Bhatti P, Conway B, Hafer N, Harter K, Kenyon N, McManus M, McNeal D, Morrato EH, Rajguru S, Wasko M. I-Corps@NCATS Trains Clinical and Translational Science Teams to Accelerate Translation of Research Innovations into Practice. *Journal of Clinical and Translational Science,* 2020, 1-64. doi:10.1017/cts.2020.561

During that time period, program adaptation for implementation science utility was initiated.

Morrato EH, McNeal D, Holtrop DA, Nearing K. The Innovation-Corps (I-Corps™) Training Program: Building D&I Capacity and Creating Stronger Value Propositions for Scaling Up and Sustaining Health Innovation. Poster presentation at the 11^th^ Annual Conference on the Science of Dissemination and Implementation in Health, Washington, D.C. December 3-5, 2018.

Nearing K, Rainwater J, Morrato EH, Neves S, Bhatti P, Hafer N, Rajguru S, Conway B,Harter K, Wasko M. I-Corps@NCATS: A novel designing-for-dissemination learning laboratory for clinical and translational researchers to increase intervention relevance and speed dissemination. Oral presentation in the Novel Approaches in D&I Training Session at the 12th Annual Conference on the Science of Dissemination and Implementation in Health, Arlington, VA December 4-6, 2019.

The program was subsequently expanded nationally to 20 CTSA hubs. One of the current authors (EHM) participated in the original train-the-trainer pilot at a previous CTSA hub (grant no. NIH/NCATS 3 UL1 TR003096-02S1). She then led implementation of the program at Loyola University of Chicago as part of the Institute of Translational Medicine CTSA hub.

The first two cohorts at Loyola Chicago were evaluated by the same national team representing three evaluation professionals with extensive CTSA evaluation experience affiliated with the UC Davis Health Clinical and Translational Science Center and the Colorado Clinical and Translational Sciences Institute, as part of the 2-year supplement. After that period the authors conducted program evaluation assisted by our local CTSA evaluation team.

To provide continuity on the evaluation outcomes, we use most of the components of the original evaluation from the national cohort. Specifically:

1. Participants experience and pathways to success with a Post survey to all participants to assess satisfaction, stage of readiness for commercialization, Collect Net-Promoter score, potential next steps. Only site-specific branding was changed. ***supplemental material II.A***
2. Fidelity of implementation- by a standardized template for documenting observations and assessing fidelity and adaptations. ***supplemental material II.B***
3. Sustainability: interviews with instructors and administrator of the program to give feedback giving important considerations that allow for quality improvement. We adapted the original interview guide for “debriefing instructors and site PIs) to fit our landscape. The interview guide is in ***supplemental material II.C***

## II.A Survey Instrument

This Qualtrics Survey instrument is administered immediately following the completion of the training program by an independent professional evaluator in the Evaluation Core of the Institute Translational Medicine CTSI.

**
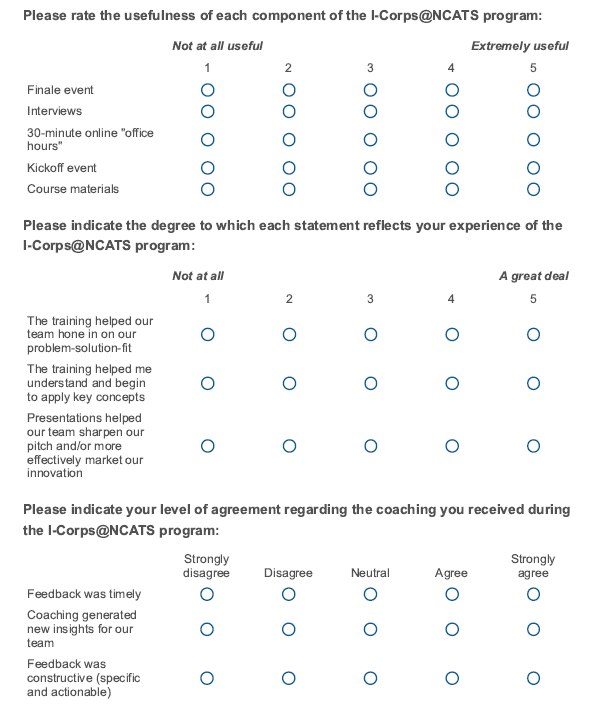
**

**
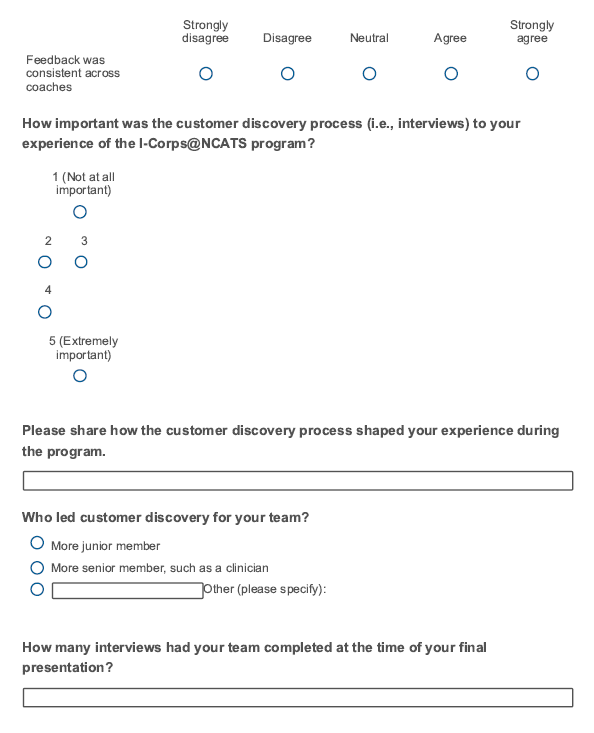
**

**
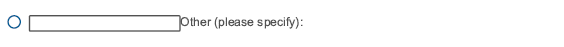

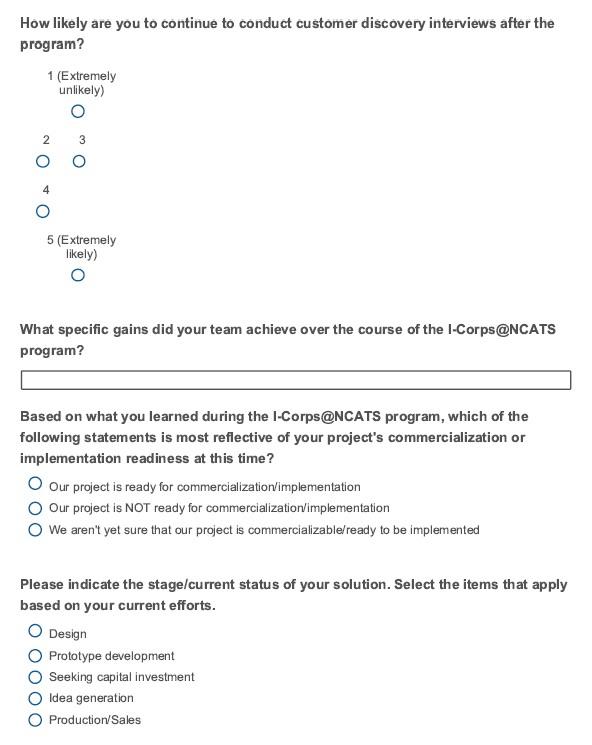
**

**
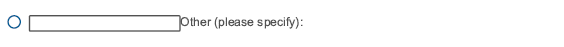
**

**
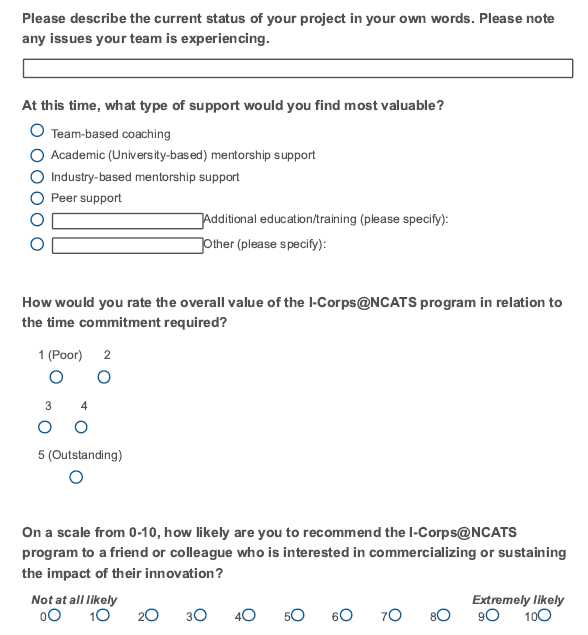
**

**
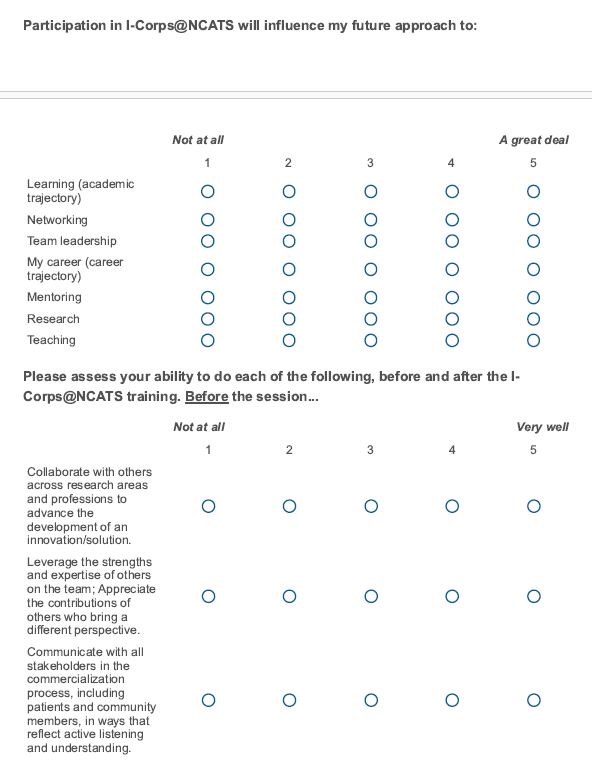
**

**
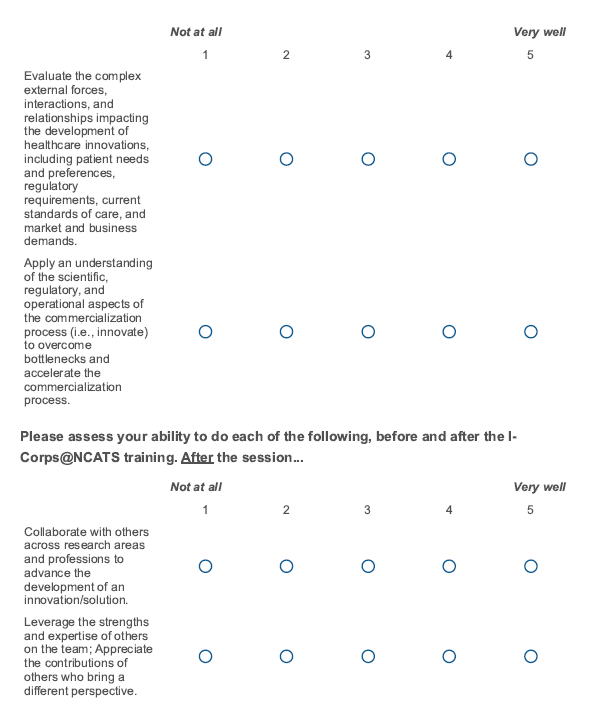
**

**
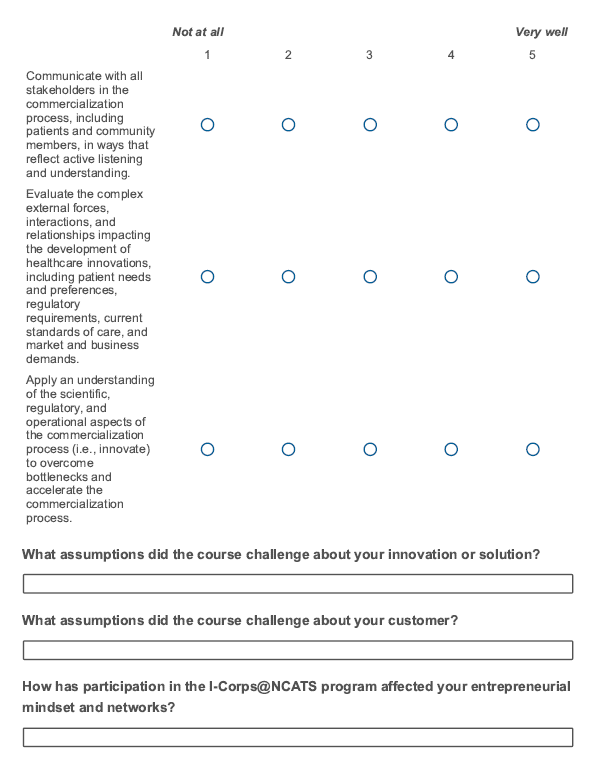
**

**
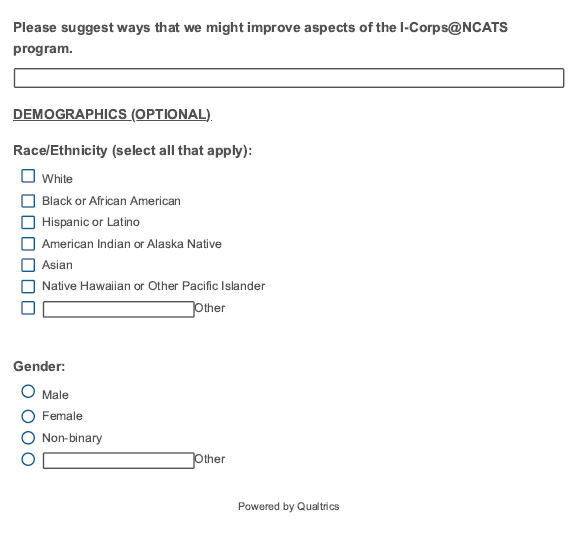
**

## II.B Observational Protocol and Fidelity-of-Implementation Checklist

This form is used to collect evidence relevant to the fidelity of implementing the I-Corps@NCATS short course at Loyola University. The information in Part One will be completed live by an observer who is a member of the evaluation team.

Goals of the fidelity assessment include addressing the following cross-site evaluation questions/ Metrics

1. Is the same information (pertaining to core concepts) being presented in the same way (e.g., with slides)?
2. How much time is spent on each topic? How variable is this across sites?
3. Are there common themes in terms of the questions that teams ask or specific aspects of content that participants seem to be more challenged by?
4. Are all intended topics covered consistently across sites?
5. Characteristics of instructional teams
6. Size of cohorts (how many teams, how many participants per team)
7. What are the ways in which the training is experiential? List key examples observed.

Background Information

1. Obtain a copy of the course syllabus and related material (e.g., pre-course reading, roster of instructors, teams).
2. How many teams participated in the kickoff?
3. On average, how many participants were on each team?
4. Did teams complete an intake survey? If yes, obtain a copy of the intake survey.
5. How many instructors participated in the kickoff?
6. How many instructors had previous experience?
7. How many instructors were local/regional trainers?

6. Describe relevant information about the classroom and facilities.

## II.C Interview guide for instructors and administrators

*I-Corps@NCATS Post-Training Faculty and Staff*

*INSTRUCTORS’ OVERALL IMPRESSIONS*

1. What were some of the unique features or aspects of the training that stand out for you? Compare/contrast cohorts.
2. Briefly, tell me about the major organizing tasks that you were involved in. What resources (time, people) did it take to carry out these tasks? What aspects of organizing a training are most labor intensive? What does it take to get all the teams to the training?
   1. What does it take to get all the teams there? Probe about: tasks, time, people resources.
3. Tell me very briefly about each of the participating teams and comment on their success, calling out the attributes/conditions that you think made them successful (and why).
   1. One aspect of the I-Corps model is the expectation that teams are comprised of at least two individuals: a senior faculty member and a more junior member, who might even be fellow or graduate student. The more junior member of the team is expected to take the lead.
      1. To what extent did you see this happening?
      2. Why is the leadership of the more junior member of the team important to the I-Corps model?
      3. What are some of the typical reasons that you might see a team struggle or have trouble during the short course?
      4. What are some of the roles of a coach in those instances?
      5. How can a coach support a team who may be struggling in that/those way(s)?
   2. As a coach, how did you work with teams behind the scenes?
   3. Do you have a story of a highlight moment as a coach that you would being willing to share?
   4. How typical is it that you might stay in touch with teams following a training?
   5. What are critical supports that you find teams need after the training?
4. I want to share a list of outcomes that we felt might be most realistic to expect of teams within the first 6 months of completing an I-Corps training. Please take a moment to scan this list.
5. Continuing customer discovery process and refining business model
6. Continuing to consult with instructors/coaches
7. Continuing to meet regularly as a team
8. Reconfiguring team in some way
9. Applying for a local CTSA funding (i.e., I-Corps seed awards, Implementation Science awards, pilot funding)
10. Applying for national I-Corps program
11. Applying for other funding (e.g., NIH SBIR)
12. Meeting with tech transfer to pursue licensing, patents
    1. Any additional outcomes not listed above that you would like to add?
    2. Which of these outcomes, including the ones you added, do you feel are most important, and why?
13. What suggestions do you have for improving the short-course model I-Corps@NCATS program? What specific features of the training would need to be modified? What resources are needed to make these changes/improvements?
